# Prognostic Role of Electrocardiographic Alternans in Ischemic Heart Disease

**DOI:** 10.3390/jcm14082620

**Published:** 2025-04-11

**Authors:** Ilaria Marcantoni, Erica Iammarino, Alessandro Dell’Orletta, Laura Burattini

**Affiliations:** Department of Information Engineering, Engineering Faculty, Università Politecnica delle Marche, 60131 Ancona, Italy; i.marcantoni@staff.univpm.it (I.M.); e.iammarino@pm.univpm.it (E.I.); s1112278@studenti.univpm.it (A.D.)

**Keywords:** ischemia, P-wave alternans (PWA), QRS-complex alternans (QRSA), T-wave alternans (TWA), percutaneous transluminal coronary angioplasty (PTCA), sudden cardiac death (SCD)

## Abstract

**Background/Objectives**: Noninvasive arrhythmic risk stratification in patients with ischemic heart disease is poor nowadays, and further investigations are needed. The most correct approach is based on the use of electrocardiogram (ECG) with the extraction of indices such as ECG alternans (ECGA). The aim of this study is to monitor the ECG evidence of ischemic coronary artery occlusion by the ECGA and to verify its ability to monitor the time course of balloon inflation, with the final goal of contributing to the exploration of the prognostic role of ECGA in ischemic heart disease. **Methods**: The ECGA amplitude and magnitude were computed by the correlation method (CM) on the STAFF III database, where ischemic coronary artery occlusion was induced in a controlled manner through coronary artery blockage by balloon inflation. ECGA computed during balloon inflation was also compared with periods before and after the inflation. **Results**: ECGA values became statistically higher during inflation than in the pre-inflation period and increased as inflation time increased, although not always in a statistically significant manner. ECGA went from values in the range 4–7 µV and 169–396 µV·beat before inflation to values in the range 5–9 µV and 208–573 µV·beat during 5 min of inflation (resulting statistically higher than before inflation), returning towards values in the range 4–8 µV and 182–360 µV·beat after inflation for amplitude and magnitude, respectively. **Conclusions**: CM-based ECGA detection was able to track the balloon inflation period. Our ECGA investigation represents a contribution in the field of research exploring its prognostic role as a noninvasive electrical risk index in ischemic heart disease.

## 1. Introduction

Ischemic heart disease refers to heart problems caused by narrowing of the cardiac arteries (coronary arteries) that supply blood to the heart muscle, due to which the heart becomes inefficient in pumping blood [1]. According to the World Health Organization (https://www.who.int/), the leading cause of death worldwide is ischemic heart disease, responsible for 13% of total deaths in 2021. In Italy, the latest news date back to 2020, with a proportion of deaths specifically due to ischemic heart disease out of the total deaths equal to 8.6%, including a higher proportion of males (9.4%) than females (7.8%). Sudden cardiac death (SCD) remains a leading cause of cardiovascular death in patients with ischemic heart disease, and ischemic heart disease accounts for 50% to 80% of SCD episodes [2]. Risk stratification is poor nowadays, and further investigations are needed to better stratify the risk of malignant arrhythmia in these patients [2]. Arrhythmic SCD has an electrical origin, and the most correct approach for arrhythmic risk stratification is based on the use of electrocardiogram (ECG), which by recording the electrical activity of the heart detects the presence and dynamics of various arrhythmogenic mechanisms [3].

Ischemia-related ECG changes may represent the only evidence of silent myocardial ischemia. Thus, extracting as much information as possible from the ECG is very important in the study of ischemic heart disease. In particular, it is necessary to find ECG-based indices that achieve high sensitivity for first-degree ischemia to promote rapid and effective intervention [4]. Conventional ECG indices, such as corrected QT interval duration (QTc), and number of ventricular premature beats, as well as advanced ECG indices such as heart-rate variability (HRV), heart-rate turbulence (HRT), and T-wave alternans (TWA), may be able to provide this prognostic information [3].

TWA is defined as the fluctuation of ECG T-wave morphology that occurs according to an ABAB pattern. The physiological origin of the phenomenon is complex and not fully understood. Some speculate that it may arise from high heterogeneity in cardiac tissue and/or voltage-dependent mechanisms and/or calcium-dependent mechanisms. The most accredited explanation is that dynamic disruptions of intracellular calcium homeostatic mechanisms occurring on a beat-to-beat basis give rise to calcium alternans. This, in turn, leads to alternans of the action potential duration (APD), as fluctuations in intracellular calcium influence repolarization dynamics [3,5]. Given the TWA association with a high risk of arrhythmic events, TWA acts as a marker of cardiac electrical instability, particularly affecting the ventricles, and is used to stratify the SCD risk [6]. In 1909, Herring reported TWA (and also QRS-complex alternans, QRSA) in animal studies, finding a connection between electrical and mechanical fluctuation, which later Lewis confirmed in human studies. Mechanical and electrical alternans have been observed in many conditions where arrhythmia is common, such as heart failure, acute or chronic myocardial ischemia, cardiomyopathy [5]. In a wider perspective, all ECG waves can be affected by the electrophysiological phenomenon of alternans. Therefore, given the possible presence (even concurrent) of P-wave alternans (PWA), QRSA, and TWA, the concept of electrical alternans has been extended to electrocardiographic alternans (ECGA).

The first method originally implemented to detect all forms of ECGA was the enhanced adaptive matched filter method (EAMFM) [7]. A limitation of its implementation core is its unsuitability for the analysis of short ECGs. In contrast, the correlation method (CM) was originally implemented to detect only TWA, but its operation can be adapted to study also the other forms of ECGA and is suitable for short ECGs.

There are several studies investigating the role of TWA in case of ischemic heart disease or ischemic heart failure, even if the conditions of observation and the methods used for the analysis were not always comparable. Kaufmann et al. observed that the prognostic value of TWA (derived from ECG applying the fast-Fourier-transform spectral method) for major adverse cardiovascular events in ischemic heart failure patients was low [8]. Duca et al. noted that TWA (analyzed by CardioScan 12 software, part of the CardioScan Holter Analysis Software suite), supplemented with other ECG-derived parameters such as HRV, can serve as a valuable diagnostic tool for the development of chronic heart failure in various diseases, but further studies are needed to confirm the prognostic value in cases where chronic heart failure is due to ischemic heart disease [6]. Cetin et al. reported the case of a patient with ischemic cardiomyopathy who had ventricular tachycardia with QRSA [9]. Vandenberk et al., in a study including patients with ischemic cardiomyopathy with a reduced left ventricular ejection fraction, found that a non-negative TWA test (analyzed by the Cambridge Heart system) was associated with increased mortality, but not with ventricular arrhythmias [10]. Rivolta et al. observed that TWA (derived from ECG applying Laplacian-likelihood-ratio method) increased progressively from the first minute to the fourth minute after the start of inflation in a population of patients undergoing coronary artery balloon occlusion angioplasty [11].

Percutaneous transluminal coronary angioplasty (PTCA) provides a useful model to study the electrophysiological changes of transmural ischemia [4]. In this context, the introduction of new procedures for the detection and characterization of transient myocardial ischemia has benefited greatly from the STAFF III database, created in the mid-1990s by the ECG acquisitions of patients undergoing prolonged elective PTCA. The complete coronary occlusion produced by balloon angioplasty made it possible to study the entire ischemic process, even the early part. ECGs acquired in conjunction with the angioplasty process are very useful in the investigation of noninvasive indices, such as ECGA.

The aim of this study is to monitor and better understand the ECG evidence of ischemic coronary artery occlusion in ECGA, with a focus on ECG-derived PWA, QRSA, and TWA identified and characterized by the CM. The ECGA was analyzed on the STAFF III database, where ischemic coronary artery occlusion was induced in a controlled manner by coronary artery blockage by balloon inflation. ECGA during balloon inflation will be also compared with resting and recovery conditions, i.e., periods before and after the inflation process. Since the predictive value of ECGA marker behavior for clinical outcome needs to be further studied and investigated [10], the ultimate goal is to make a contribution to the exploration of ECGA prognostic role in relation to the electrical dynamics of ischemic heart disease.

## 2. Materials and Methods

### 2.1. Study Population Description

The STAFF-III database includes 108 patients who were admitted to the Charleston Area Medical Center in West Virginia, USA, in 1995 and 1996, for elective prolonged PTCA since they were affected by stable angina pectoris [12]. Even if the previous medical history was unknown, all patients were clinically stable during the study protocol. In particular, there were no ventricular abnormalities, emergency procedures, or signal loss either at the time of inclusion or during the surgical procedure [13]. The treatment protocol involved a single (or multiple) prolonged ballon inflation(s), lasting between 1.5 and 9.9 min, with a mean of 4.4 min [13]. Overall, the locations of the occlusion along the major coronary arteries were different among patients and they could affect the left anterior descending artery (LAD), the right coronary artery (RCA), and the left circumflex artery (LCX). This information was specified in the annotations.

The study protocol included the continuous acquisition of 9-lead (V1–V6, I, II, and III) ECG recordings by the use of a custom-made recording equipment provided by Siemens-Elema AB (Solna, Sweden; sampling rate: 1000 Hz; amplitude resolution: 0.6 mV) [13,14]. Precordial leads were acquired according to the standard electrode placement, while the limb leads followed the Mason–Likar electrode configuration [13]. The three augmented leads aVL, aVR and aVF were computed from the limb leads to generate the complete standard 12-lead ECG. ECG was acquired before, during, and after PTCA.

**Figure 1:**
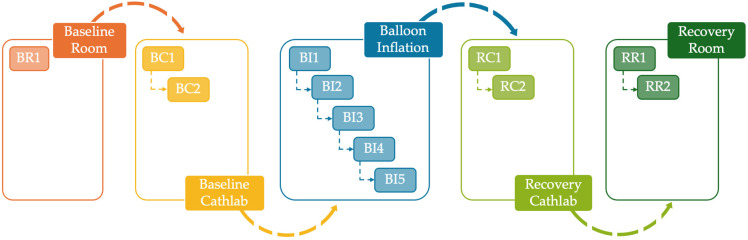
The sequence of recording conditions. Each color is associated with a different protocol phase among the three considered (baseline, inflation, recovery).

ECG signals were acquired before inflation (baseline) for 5 min at rest in a supine position, either in a relaxing room (baseline room, up to one time per patient), catheterization laboratory (baseline cathlab, up to two times per patient), or both, before catheter insertion. ECG recordings during balloon inflation were acquired up to five times per patient. They were continuously acquired from about 1 min before the inflation until about 4 min after deflation. ECG recordings after balloon inflation were acquired for 5 min, either at rest in supine position in a catheterization laboratory (recovery cathlab, up to two times per patient), relaxing room (recovery room, up to two times per patient), or both. The sequence of recording conditions is graphically outlined in Figure 1. Overall, the database includes a minimum of two ECG acquisitions to a maximum of seven ECG acquisitions per patient.

The time instants of balloon inflation and deflation were specified in the annotations. In some cases, patients underwent multiple balloon inflations, and for each, a separate acquisition protocol was performed as independent. Enrolled patients previously provided informed consent. The study was approved by the local Investigational Review Board [13,14].

For four patients (specifically, those numbered as 28, 67, 78, 103), the raw data and metadata were not present in the database, without any specification by the database creators, with a total of 104 patients. Among them:

- Less than 35%, 10%, 2%, and 1% of patients had two, three, four and five ECG acquisitions during balloon inflation, respectively;
- More than 70% of patients had the ECG acquisition in the baseline room;
- More than 99% and 10% of patients had one and two ECG acquisitions in the baseline cathlab, respectively;
- About 90% and 2% of patients had one and two ECG acquisitions in the recovery cathlab, respectively;
- More than 90% and 2% of patients had one and two ECG acquisitions in the recovery room, respectively.

For five patients (specifically, those numbered as 1, 4, 5, 6, 89), some derivations of the recordings were later identified by the database creators as incorrect. Thus, we decided to exclude them from the enrolled study population, with a total of 99 remaining patients.

Basic information about gender, age, pre-occlusion heart rate and occlusion time were available among metadata. We found that some patients had information about occlusion times that was discordant with that obtainable from the raw data. In these cases, if multiple balloon inflations were performed, only the raw data in agreement with the annotations were retained, otherwise the patient was not included in the study population. Based on these exclusion criteria, an additional 7 patients were not included in the study population, resulting in a study population of 92 patients, with 33 females and 59 males, aged between 32 and 100 years, of which 58 underwent a single balloon inflation [13,14].

### 2.2. Recursive Extraction of Electrocardiographic Windows and Preprocessing

A preprocessing phase of raw ECGs preceded the identification and quantification of ECGA. Firstly, ECGs were down sampled at 200 Hz. Then, for each available ECG, overlapping and sliding windows containing 32 heartbeats were recursively (every second) extracted until the end of the signal was reached. All leads inside the ECG window were low-pass filtered by means of a 6th-order bidirectional Butterworth filter having a cut-off frequency of 45 Hz. The baseline wandering was reconstructed using a cubic spline interpolation of reference points located 80 ms before the R peaks. The R peaks were also used to compute the RR interval series (ms), and then the related mean and standard deviation, as an estimation of HRV.

### 2.3. Identification and Synchronization of Electrocardiogram Sections

In each heartbeat, three ECG sections were identified: the section including the P wave, the section including the QRS complex, and the section including the T wave. The limits of these ECG sections, i.e., the onsets (Pon, Qon, and Ton) and the offsets (Poff, J, and Toff), were detected through the application of formulas derived experimentally, usually based on RR (Table 1).

For each ECG window, the ECG section underwent a synchronization step. The synchronization procedure was based on the correlation of ECG sections with the template, where the template is the median of all ECG sections included in the ECG window considered. Specifically, each ECG section was aligned with the template by finding the maximum correlation (quantified through the Pearson’s coefficient) when the ECG section was translated within a window between −0.03 s and +0.03 s around the limits found according to Table 1. The alignment of each ECG section with the same template resulted in the synchronization of all ECG sections with each other.

### 2.4. Electrocardiographic Alternans Detection Through the Correlation Method

The CM was the method used to identify and quantify ECGA, whose implementation details can be found in [15]. The CM was applied to ECG windows and leads that met both the suitability criteria, i.e., two conditions to verify RR stability and sinus rhythm. As for the first criterium, the ECG window was rejected if RR standard deviation overcame 10% of the mean RR. As for the second criterium, the specific lead of the ECG window was rejected if both QRS and T sections correlated with the related template less than 80% for more than two heartbeats. Otherwise, only these heartbeats were replaced with a median heartbeat computed over all the present ones, without rejecting the specific lead of the ECG window. The rejection implied not analyzing the ECG window tracing and not assigning any value to the feature extracted for the ECGA characterization.

For each ECG window, CM was applied three times independently considering the synchronized P, QRS and T sections. Starting from the ECG sections and the related templates (i.e., median P section, median QRS section, and median T section), an alternans correlation index (ACI) was computed according to Equation (1):

(1) $${ACI}_{j}=\frac{\sum_{i=1}^{N_{S}}S_{j}\left(i\right)\cdot S_{mdn}(i)}{\sum_{i=1}^{N_{s}}{S_{mdn}}^{2}(i)},$$

where j ranged from 1 to N_T_ (N_T_: number of heartbeats in the ECG window), N_S_ was the number of samples in the heartbeat section considered, S_j_ was the heartbeat section considered, and S_mdn_ was the related median (template). ACI quantified the maximum cross-correlation function of the heartbeat section vs. the template over the auto-correlation function of the template. ACI could range around 1 if ECGA was monophasic or around 0 if ECGA was biphasic. ECGA was identified for each heartbeat belonging to a series of five consecutive alternating heartbeats. Otherwise, ECGA was considered absent and a value of 0 µV was assigned to the heartbeat. In case of ECGA identification, its amplitude was computed according to Equation (2):

(2) $$A_{CM}\left(j\right)=2\left|{ACI}_{j}-1\right|\;\cdot \;\frac{\sum_{i=1}^{N_{S}}{S_{mdn}}^{2}(i)}{\sum_{i=1}^{N_{s}}\left|S_{mdn}(i)\right|}$$

The output of CM was the ECGA heartbeat-related amplitudes (A_CM_, µV). Thus, from the three applications, a quantification of PWA, QRSA, and TWA amplitudes was obtained in each ECG window per heartbeat and per lead.

### 2.5. Statistics

#### 2.5.1. Single-Subject Level

In each ECG window and from each available lead, the following features were extracted:

- The PWA, QRSA, and TWA local amplitudes (µV), computed as the median PWA, QRSA, and TWA amplitudes over all heartbeats;
- The PWA, QRSA, and TWA local magnitudes (µV·beat), computed as the median product of PWA, QRSA, and TWA amplitudes over the heartbeats with an amplitude greater than 0 µV, and their amount (which accounts for the duration of the ECGA episode, and expressed as number of heartbeats).

Then, among the available ECG leads, only the maximum value of each ECGA-related feature was retained. Since the ECG windows were recursively extracted every second, the trends of PWA, QRSA, and TWA amplitudes and magnitudes sampled at 1 Hz were obtained per patient, for the baseline, inflation and recovery phases.

In the baseline phase, the trends obtained only in the ECG recorded in the baseline room or, if it was not available, the ECG recorded in the baseline cathlab were considered. Analogously, in the recovery phase, the trends obtained only in the ECG recorded in the recovery room or, if it was not available, the ECG recorded in the recovery cathlab were considered. For both the baseline and recovery phases, the first and last 10 s were excluded.

For the inflation phase, the trends obtained from all the ECGs recorded in the inflation phase were considered. This phase was divided into three subphases: the one before inflation; the one during inflation; the one after inflation. According to the duration of the inflation, the trends were classified into three categories: (1) inflations lasting less than 150 s; (2) inflations lasting between 150 s and 250 s; (3) inflations lasting more than 250 s.

#### 2.5.2. Multi-Subject Level

For each phase, the median trends (together with their interquartile ranges) over the population were computed, so to obtain comprehensive trends of PWA, QRSA, and TWA amplitudes and magnitudes. For the inflation phase, the median calculation was preceded by synchronization of the inflation subphases (taking the beginning of the subphase as the reference time instant), and the considered durations of the subphases before and after inflation were the median durations over the population included in the specific category. Finally, all trends were smoothed using a moving average filter.

In addition, for all the patients, the median ECGA amplitude and magnitude were computed for both the baseline and recovery phases, while the maximum ECGA amplitude and magnitude were extracted for the inflation phase. These three population distributions in the phases before, during, and after inflation were compared in all the possible combinations for the same feature kind (i.e., amplitude and magnitude) through the Wilcoxon rank sum test, setting the statistical difference (*p*) at 0.05. The same comparisons were performed considering only the patients having a single balloon inflation and a correspondent ECG recording in the baseline and recovery phases, so that this time, a Wilcoxon signed rank test was applied.

Referring to the longest inflation duration category, the 25th, 50th (median) and 75th percentiles of PWA, QRSA and TWA amplitudes and magnitudes were calculated over the included population minute by minute, monitoring the temporal trend of inflation. Each consecutive 1 min interval within the inflation was compared with the baseline phase and, starting with the second 1 min interval, also with the previous 1 min interval. Comparisons were performed by Wilcoxon rank sum test, setting the statistical difference (*p*) at 0.05.

## 3. Results

Overall, the number of ECG recordings analyzed in the baseline phase was 92 (RR = 918 [778; 1015] ms, expressed as 50th [25th; 75th] percentiles, as in the following), in the balloon inflation phase, it was 120 (RR = 847 [723; 930] ms), and in the recovery phase, it was 90 (RR = 843 [710; 957] ms). Considering the three categories defined according to the duration of the inflation, we found that (1) in the first category, the number of ECG recordings included was 17 (RR = 869 [800; 963] ms) and the duration of the balloon inflation was 117 ± 17 s; (2) in the second category, the number of ECG recordings included was 29 (RR = 839 [718; 921] ms) and the duration of the balloon inflation was 194 ± 25 s; (3) in the third category, the number of ECG recordings included was 74 (RR = 830 [710; 957] ms) and the duration of the balloon inflation was 306 ± 42 s.

**Figure 2:**
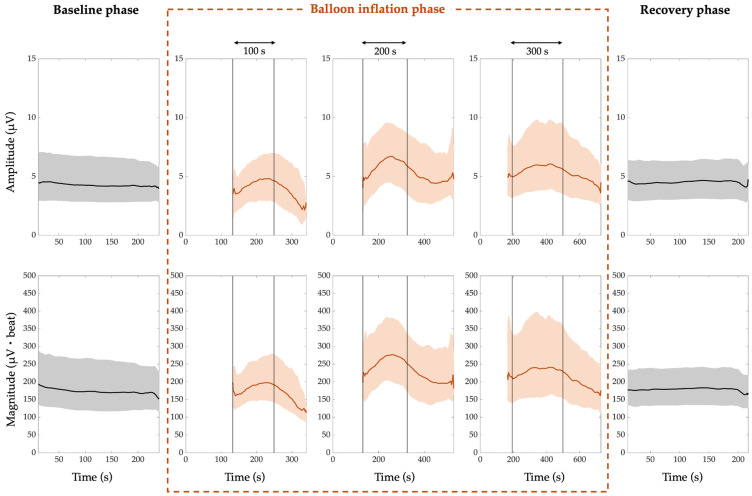
P-wave alternans (PWA) trend. The amplitude trends are shown in the first row, while the magnitude trends are shown in the second row. The 50th percentile is represented by thick lines, and the interquartile range is represented by shaded areas, while the vertical lines mark the beginning and end of the inflation process.

**Figure 3:**
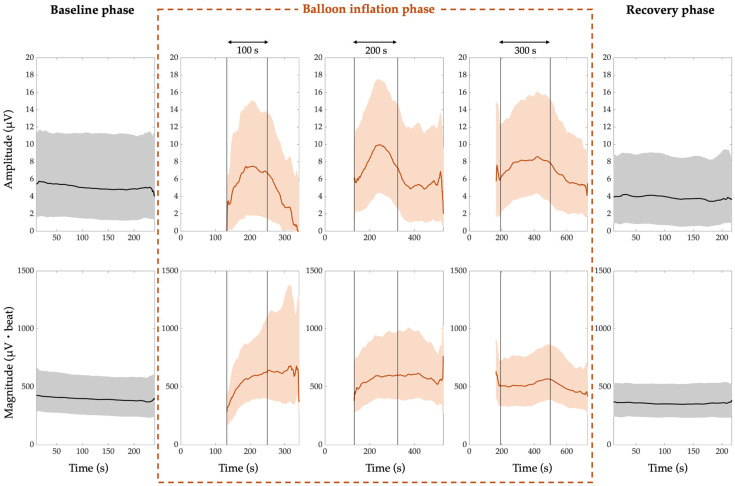
QRS-complex alternans (QRSA) trend. The amplitude trends are shown in the first row, while the magnitude trends are shown in the second row. The 50th percentile is represented by thick lines, and the interquartile range is represented by shaded areas, while the vertical lines mark the beginning and end of the inflation process.

**Figure 4:**
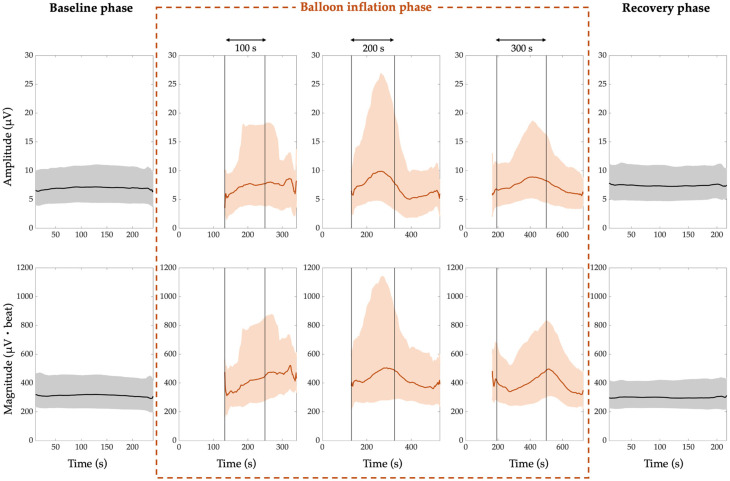
T-wave alternans (TWA) trend. The amplitude trends are shown in the first row, while the magnitude trends are shown in the second row. The 50th percentile is represented by thick lines, and the interquartile range is represented by shaded areas, while the vertical lines mark the beginning and end of the inflation process.

The comprehensive amplitude and magnitude trends of PWA, QRSA, and TWA are shown in Figure 2, Figure 3, and Figure 4, respectively. In all the figures, the amplitude trends are reported in the first row, while the magnitude trends are reported in the second row. Moreover, the 50th percentile (median) is depicted by thick lines, which are black for the trends referring to the resting and recovery phases and red for the trends referring to the balloon inflation phase. The interquartile range is depicted by shaded areas, of which the lower and upper contours are the 25th and 75th percentiles, respectively. The vertical lines in the balloon inflation panels indicate the median time points of the beginning and end of the inflation process, since the ECG recordings in this phase also include the periods immediately before and immediately after (subphases before and after inflation). For simplicity, the three different categories of balloon inflation duration are indicated by rounding to the nearest hundredth seconds. The white zones before the balloon inflation time interval account for the maximum duration of the periods immediately before the balloon inflation. Indeed, the within-inflation periods (inflation subphases) are completely covered by the trends, while the periods immediately before and after (subphases before and after inflation) are covered only for the median duration, and the subphase before inflation was very often not present in the ECG recordings.

From the results, we can notice that the trends related to the baseline and recovery phases are almost flat. Indeed, there is an inter-subject variability, quantified by the grey areas, but the time variability is very low.

For the baseline phase:

- The median PWA, QRSA, and TWA amplitude trend is around 4 µV, 5 µV, and 7 µV, respectively;
- The median PWA, QRSA, and TWA magnitude trend is around 169 µV·beat, 396 µV·beat and 310 µV·beat, respectively.

For the recovery phase:

- The median PWA, QRSA, and TWA amplitude trend is around 5 µV, 4 µV, and 8 µV, respectively;
- The median PWA, QRSA, and TWA magnitude trend is around 182 µV·beat, 360 µV·beat and 296 µV·beat, respectively.

Thus, in the recovery phase, the ECGA seems to return to the baseline values.

In the balloon inflation phase, the trends are always increasing from the beginning of the process, and then lean towards the recovery values almost immediately after the end of the process, except for the QRSA in the shortest and intermediate inflation duration categories and for the TWA in the shortest duration category.

The median ECGA amplitude and magnitude were not statistically different between the baseline and recovery phases (*p* > 0.05). The maximum ECGA amplitude and magnitude were statistically different from both the baseline and recovery phases (*p* < 10^−8^). When considering patients with single balloon inflation, the same results were reached, except for QRSA amplitude and magnitude, which were statistically different also when comparing baseline and recovery phases (*p* = 0.04).

Table 2 reports minute by minute courses for 25th, 50th (median) and 75th percentiles of PWA, QRSA and TWA amplitudes and magnitudes for the longest inflation duration category. Most (90%) of the 1 min intervals, characterized in terms of PWA, QRSA, and TWA amplitude and magnitudes, were statistically different with respect to the baseline phase, while only the QRSA amplitude computed in the second 1 min interval and the TWA amplitude computed in the third 1 min interval resulted statistically different with respect to the preceding 1 min intervals.

## 4. Discussion

The presented study analyzed the behavior of ECGA in a population of patients undergoing elective PTCA, a procedure belonging to the pre-stent era. The study population is included in the STAFF III database. The analysis was possible since before, during and after the procedure, high time-resolution 12-lead ECGs were acquired, and the raw data, along with the metadata, were made freely available on PhysioNet, a repository managed by the MIT Laboratory for Computational Physiology, including medical research data [16]. Therefore, we considered it the most suitable database to test the ability of the CM-based ECGA to track the time course of balloon inflation in coronary arteries to induce an ischemic condition for the heart, also comparing it with the baseline and recovery values.

As just mentioned, the CM was chosen for the analysis [15]. Many methods are present in the literature to detect TWA, and two of them, the modified moving average [17] and the fast-Fourier-transform spectral [18] methods, are also commercially available. Nevertheless, previous research comparing the CM with the enhanced version of the modified moving average method [19] verified that the two methods are equivalent when analyzing ECGs free of artifacts or noise superimposed on the useful signal and influenced by stationary TWA. On the contrary, the modified moving average method, even in its enhanced version, is influenced by the presence of interferences or other types of variability that are erroneously associated with the TWA, thus generating false positives. In addition, the non-stationary TWA is correctly identified by the CM, but not by the modified moving average and the fast-Fourier-transform spectral methods [20,21]. All the methods present in the literature, except for the enhanced adaptive matched filter [7], were originally implemented to study TWA, the most analyzed form of ECGA; thus, CM implementation was properly adjusted to extend its output from TWA alone to ECGA, including also PWA and QRSA.

The preprocessing performed here on the ECGs, which involved not only digital filtering and baseline removal but also the analysis of suitability criteria, although implying the rejection of some ECG windows and/or leads, ensured a reliable analysis. In fact, the stable RR criterion allowed us not to interpret variability of different nature as ECGA, and the sinus rhythm criterion prevented the inclusion of portions of the ECG trace influenced by artifacts.

Two features were used to characterize ECGA forms: amplitude and magnitude. The amplitude gives an overall evaluation of the phenomenon entity, where all the heartbeats, even if not recognized as alternating, are included. In contrast, the magnitude includes only the alternating heartbeats and accounts also for the duration of the episodes. Moreover, in its original implementation, the ECGA identification criterion used by CM was arbitrarily set to detect seven consecutive alternating heartbeats within an ECG window containing 128 heartbeats. Given the short temporal duration of the ECG windows considered here (32 heartbeats), which in turn is conditioned by the duration of balloon inflation and the need to extract several ECG windows to better isolate the ECG portions affected by noise, we reduced this threshold to five. We decided to use the maximum value of alternans among the available ECG leads in accordance with [6], so as to maintain only the ECG lead that best highlights the electrophysiological phenomenon of the ECGA, which is lead-dependent [22].

The obtained results showed that all ECGA forms were able to track the time course of balloon inflation inducing cardiac ischemia, by rising (even if not always statistically) from the beginning of process and starting to decrease only at the end of the process. This observation can be justified by the fact that chronic ischemia can elicit alterations in ionic currents in cardiac cells, mainly involving calcium and potassium ions, resulting in beat-to-beat depolarization and/or repolarization variability, which manifests on ECG as alternans [6]. In addition, chronic ischemia is listed among the disease conditions that impair calcium cycling at physiological heart rates [5]. The canonical examples of arrhythmogenic disorders of early repolarization are Brugada syndrome and short QT syndromes. However, early repolarization, known to be associated with TWA, has also been reported in acute myocardial ischemia [5,23].

ECGA trends change depending on how long the occlusion lasts. More specifically, while the general shape or direction of the patterns—rising and falling—remains the same, the timing of these changes differs based on the duration of the occlusion. So, the trends follow a similar rise-and-fall pattern, but how quickly or slowly they occur depends on how long the blockage lasts. This observation is coherent with our initial hypothesis that led us to organize the analysis into three distinct groups, with each group categorized according to how long the occlusion lasted. The varying timings for alternans to reach its peak and subsequently decline in our study were likely primarily influenced by the occlusion timing set by the database creators defining the protocol. Nevertheless, we have to consider that the literature suggests that different occlusion durations can result in heterogeneous timing for the onset of ischemic effects, such as ion imbalances and increased APD dispersion. Notably, previous studies examining prolonged artery occlusions, even if focused solely on TWA, have observed a similar trend. For example, Martínez et al. [24], using an animal model, observed a monotonic increase in TWA amplitude during the first 300 s of ischemia, followed by a significant decline between 300 and 360 s. The underlying electrophysiological mechanism relies on the dynamics of APD during ischemia. Ueda et al. [25] demonstrated that arterial occlusion initially prolongs APD for approximately 240 s, after which it begins to shorten. Bernikova et al. [26] further explained that this transient APD prolongation is the first electrophysiological change observed in ischemia, primarily due to the suppression of potassium currents, in agreement with the QTc interval lengthening seen in the early minutes of coronary occlusion in pigs. Similarly, Burton and Cobbe [27] reported studies confirming that ischemia-induced APD prolongation occurs within the first 120 s of coronary occlusion. This phase is then followed by APD shortening, attributed to a progressive reduction in resting membrane potential, action potential amplitude, and depolarization velocity. The mechanisms behind it were further clarified by Oknińska et al. [28], who described how, in the early minutes of acute ischemia, potassium channel conductance is blocked, while a sustained influx of sodium ions prolongs the plateau phase and thus APD. However, as ischemia progresses, the activation of potassium channels and intracellular accumulation of sodium and calcium contribute to APD shortening.

Evidence suggests that states of high ECGA are associated with an unstable electrical substrate that degenerates into arrhythmia when a trigger occurs, such as a premature impulse. This paradigm would indicate that high levels of ECGA may have a determining clinical role as predictors of tachycardia or fibrillation [29]. In this context, we could also hypothesize a relationship between the extent of the electrophysiological phenomenon of ECGA, the entity of the ischemic condition, and the probability that degeneration into arrhythmia occurs, which would give an even more significant role to ECGA in defining the severity of the ongoing ischemic phenomenon. Thus, the investigation carried out in this study paves the way for an effective arrhythmic risk stratification approach based on ECGA, which however should be further investigated, also considering longer occlusions than the ones considered in the present work.

In our analysis, it would have been useful to take into consideration possible prior cardiac artery disease inducing ischemic preconditioning. Ischemic preconditioning refers to a brief episode of ischemia causing endogenous cardioprotection, making the heart more resistant during a subsequent prolonged episode. In the context of PTCA, clinical observations have shown that ischemia evidence, including the ECG-derived one, occurring during the artery occlusion by balloon inflation decreases during a second balloon inflation [30]. Therefore, as cardiac preconditioning could affect the evaluation of our results, it should be pointed out that we could not consider this possible confounding factor due to the lack of prior clinical information about the patients, as well as due to the small amount of multiple inflations, which represents a first limitation of the analyzed database. Recognizing another limitation of the study and of the database considered, it is necessary to note that we did not distinguish among ECGs related to different occluded arteries and we treated them the same in the analysis. This stratification was not feasible due to the small size of the resulting subpopulations, which would not allow us to achieve statistical significance and thus conclusive results. The lack of stratification based on location and extent of ischemia might be the reason why we did not observe very different behavior among the different forms of ECGA.

Further investigations involving wider populations should assess the PWA, QRSA, and TWA dynamics in relation to the extent of arterial occlusion and its specific anatomical localization, so to identify ECG-derived indices or different behaviors of the same index, depending on the specific condition. Previous studies have focused only on TWA even in the case the anomaly (such as artery occlusion) affected atria rather than ventricles; thus, the novelty of our study lies in focusing on the other forms of ECGA as well. This approach would provide further insights into the pathophysiological link between the extent and severity of the ischemic condition and noninvasive ECG-based arrhythmic risk stratification. In addition, investigating the ECGA role in ischemia would be particularly useful in guiding therapeutic decisions and clinical management, and in monitoring the effects of ischemia treatment and reperfusion, helping to assess the extent of myocardial damage or recovery.

## 5. Conclusions

The aim of this study was to monitor ECG evidence of ischemic coronary artery occlusion by ECGA, discriminating among different forms of ECGA (i.e., PWA, QRSA, and TWA) and two different features (i.e., amplitude and magnitude). CM-based ECGA detection appeared to be able to track the balloon inflation period within the first minute for PWA, within the second minute for QRSA, and within the third minute for TWA. ECGA values became statistically higher during inflation than in the pre-inflation period and increased as inflation time increased, although not always in a statistically significant manner. ECGA investigations, such as the one performed here, provide a contribution in the field of research exploring its prognostic role as a noninvasive electrical risk index in ischemic heart disease.

## Figures and Tables

**Table 1:** Identification of electrocardiographic sections: Formulas to detect the limits.

Section ^1^	Onset ^2^	Offset ^2^
**P section**	(a) if RR < 0.6 s, Pon = Qon − 0.18 s (b) if 0.6 s < RR < 1.1 s, Pon = Qon − 0.2 s (c) if RR > 1.1 s, Pon = Qon − 0.25 s	$Poff=Pon+0.2\cdot \sqrt{mRR}$
**QRS section**	Qon = R − 0.05 s	J = R + 0.05 s
**T section**	(a) if RR < 0.6 s, Ton = R + 0.06 s (b) if 0.6 s < RR < 1.1 s, Ton = R + 0.10 s (c) if RR > 1.1 s, Ton = R + 0.15 s	(a) if RR < 0.6 s, Toff = Ton + 0.35 ·$\sqrt{mRR}$ (b) if 0.6 s < RR < 1.1 s, Toff = Ton + 0.4 s (c) if RR > 1.1 s, Toff = Ton + 0.45 s

^1^ P section: section including the P wave; QRS section: section including the QRS complex; T section: section including the T wave. ^2^ R: R peak position(s); mRR: mean RR interval(s).

**Table 2:** Minute-by-minute trends of ECGA in 300 s long balloon inflation category. ECGA is reported as PWA, QRSA, and TWA amplitudes and magnitudes, expressed as 50th [25th; 75th] percentiles.

ECGA		Baseline	Balloon Inflation				
Form	Feature		1st Minute	2nd Minute	3rd Minute	4th Minute	5th Minute
**PWA**	A ^1^ (µV)	4[3;7]	5*[4;7]	6*[4;9]	6*[4;9]	6*[4;10]	6*[4;11]
	M ^2^ (µV·beat)	169[126;250]	208*[154;328]	237*[160;382]	239*[161;389]	234*[171;398]	240*[172;385]
**QRSA**	A ^1^ (µV)	5[2;9]	6[3;11]	8*^,†^[4;14]	8*[5;15]	9*[5;15]	9*[5;13]
	M ^2^ (µV·beat)	396[258;528]	511*[350;756]	523*[355;720]	508*[356;756]	573*[366;805]	552*[437;790]
**TWA**	A ^1^ (µV)	7[5;9]	7[5;10]	7[5;11]	9*^,§^[6;13]	9*[6;18]	9*[6;18]
	M ^2^ (µV·beat)	310[234;404]	376*[264;558]	328*[260;486]	396*[271;613]	416*[278;724]	460*[270;724]

^1^ Amplitude; ^2^ magnitude. *: *p* < 0.05 comparing against the baseline; ^†^: *p* < 0.05 comparing against the 1st minute; ^§^: *p* < 0.05 comparing against the 2nd minute; ^‡^: *p* < 0.05 comparing against the 3rd minute; ^$^: *p* < 0.05 comparing against the 4th minute.

## Data Availability

The data used in the study are openly available in PhysioNet (https://physionet.org/content/staffiii/1.0.0/) (accessed on 1 October 2024).

## Abbreviations

The following abbreviations are used in this manuscript:

APD	Action Potential Duration
CM	Correlation Method
ECG	Electrocardiogram
ECGA	Electrocardiographic Alternans
HRT	Heart-Rate Turbulence
HRV	Heart-Rate Variability
PTCA	Percutaneous Transluminal Coronary Angioplasty
PWA	P-Wave Alternans
QRSA	QRS-complex Alternans
SCD	Sudden Cardiac Death
TWA	T-Wave Alternans
